# The diagnostic role of machine learning models related to visceral fat with sex-specific distribution in subtypes of primary aldosteronism

**DOI:** 10.7150/ijms.131815

**Published:** 2026-04-23

**Authors:** Yu Luo, Xiaoyu He, Fen Wang, Luoning Gou, Zheng Liu, Chuou Xu, Anhui Xu, Junhui Xie

**Affiliations:** 1 Division of Endocrinology, Tongji Hospital, Tongji Medical College, Huazhong University of Science and Technology, Branch of National Clinical Research Center for Metabolic Diseases, Hubei, China.; 2 Department of Urology, Tongji Hospital, Tongji Medical College, Huazhong University of Science and Technology, Hubei, China.; 3 Department of Radiation Technology, Tongji Hospital, Tongji Medical College, Huazhong University of Science and Technology, Hubei, China.

**Keywords:** Primary aldosteronism, cardiometabolic risk, Visceral fat deposition, Machine learning model

## Abstract

**Background:**

Primary aldosteronism (PA) subtypes exhibit significant sex-specific differences, particularly regarding cardiovascular risk and the metabolic syndrome. This study investigated these differences between subtypes and developed a machine learning model for subtype prediction.

**Design and Methods:**

This retrospective study analyzed clinical and imaging data from 276 PA patients, subtyped by adrenal venous sampling. Visceral and cardiac adipose deposition were quantified, least absolute shrinkage and selection operator (LASSO) regression identified optimal predictive parameters for model development.

**Results:**

Unilateral PA (UPA) presented with prominent hypertension and hypokalemia, whereas bilateral PA (BPA) showed more visceral fat deposition, with notable sex differences. (1) In males, the BPA group had a higher body mass index, abdominal fat, and larger epicardial adipose tissue (EAT) volume along with a greater E/e' ratio compared to UPA. (2) Across both subtypes, males demonstrated more abdominal fat than females. (3) In females, BPA had higher triglycerides, serum calcium than UPA. (4) In males, an XGBoost model using visceral fat area (VFA), serum potassium, systolic blood pressure, and plasma aldosterone concentration (PAC) achieved an AUC of 0.84±0.05. An XGBoost model in females based on serum potassium, lipid profile, PAC after saline infusion test, and CT nodule characteristics yielded an AUC of 0.75±0.02.

**Conclusion:**

PA subtypes exhibit prominent sex-specific cardiometabolic profiles. In males, BPA correlates with significantly greater ectopic fat deposition and elevated cardiovascular risks. Combining VFA with key biochemical markers demonstrates superior diagnostic efficacy for PA subtyping in the male group.

## 1. Introduction

Primary aldosteronism (PA) is characterized by excessive aldosterone production independent of renin, manifesting clinically as hypertension and hypokalemia 1, 2. Multiple studies 3, 4 have demonstrated the prevalence of 5%-15% for PA among hypertensive patients. Unilateral PA (UPA) and bilateral PA (BPA) are two major subtypes of PA. It is important to distinguish them for proper treatment, as they influence management decisions such as surgical intervention or medical therapy. Adrenal venous sampling (AVS) is the gold standard for PA subtyping but is invasive, technically complex, and not widely available. Machine learning models have emerged as a promising alternative for PA subtype prediction. However, current machine learning approaches require further personalization and precision for optimal subtype differentiation. Noninvasive diagnosis of PA subtypes remains a challenge in clinical practice. Visceral fat deposition has been shown to vary between PA subtypes, with sex-specific differences in clinical presentation, cardiovascular risk, and metabolic comorbidities such as diabetes and osteoporosis 5-10. These patterns suggest visceral fat may serve as a useful biomarker for subtype differentiation.

This study investigated sex-specific differences in metabolic characteristics and cardiovascular risk, particularly visceral fat deposition, between the two PA subtypes. It also seeks to develop sex-specific machine learning models to improve clinical subtyping accuracy.

## 2. Methods

### 2.1 Study Design

**Figure 1:**
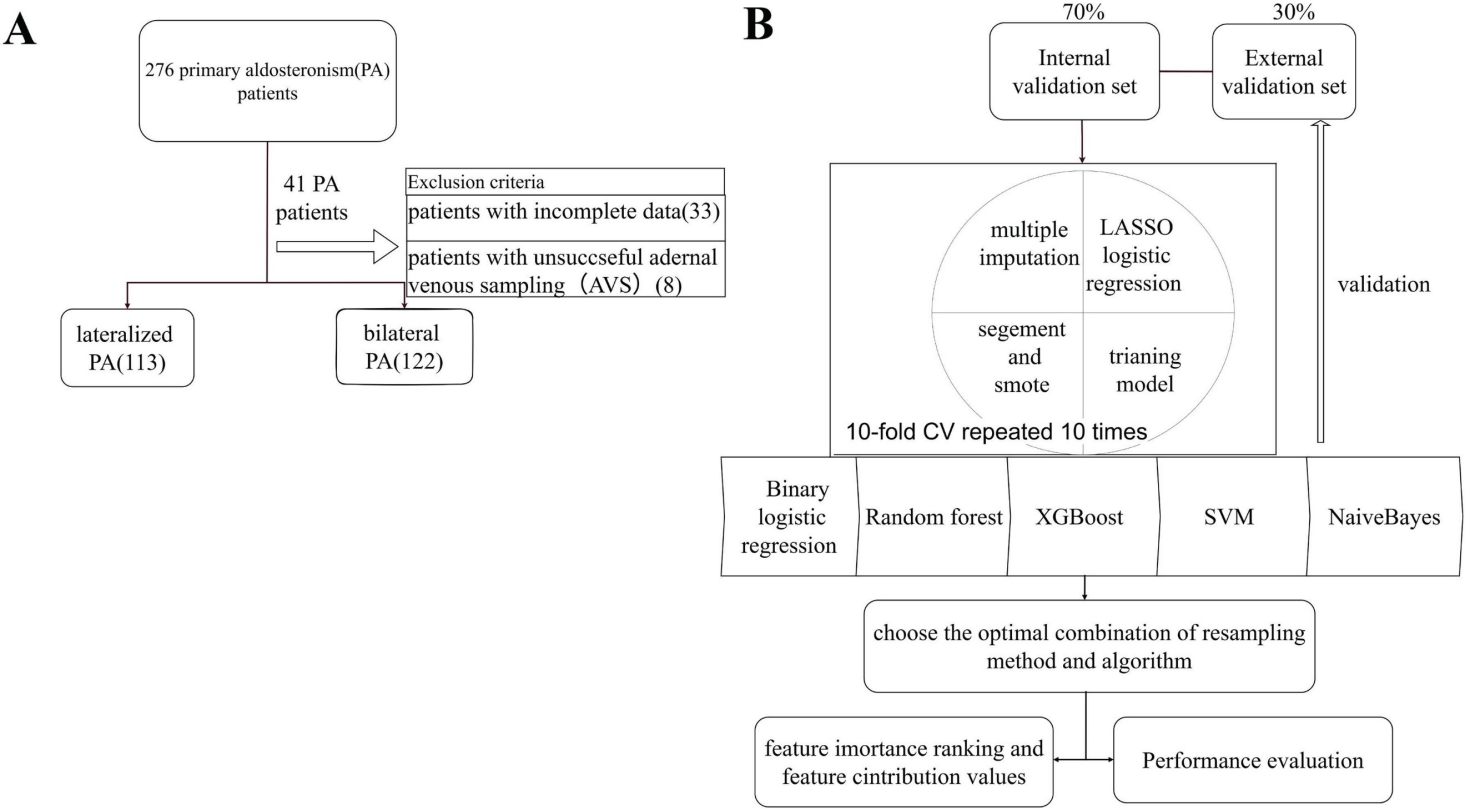
** Workflow and predictive model setup procedure. (A)** The overall study workflow. **(B)** The predictive model setup procedure. Lasso: least absolute shrinkage and selection operator; CV: cross-validation; XGBoost: extreme gradient boosting; SVM: support vector machine; ROC: receiver operating characteristic curve.

This retrospective study enrolled 276 patients with PA who visited the Tongji Hospital, Tongji Medical College, Huazhong University of Science and Technology, across three campuses between 2014 and 2024. Patients with unsuccessful AVS or incomplete data were excluded. Biochemical and imaging parameters were collected and measured for machine learning analysis to develop a predictive model for PA subtypes (Figure 1). Diagnosis was confirmed using positive confirmatory tests like captopril challenge test (CCT) or saline infusion test (SIT) according to the 2016 AACE guidelines 11. Patients were categorized into BPA and UPA groups based on AVS results 12. The selectivity index (SI), lateralization index (LI), and central index (CI) were calculated (Supplementary Material).

### 2.2 Data Collection

#### 2.2.1 Clinical and Laboratory Data

Demographic data such as age, sex, weight, height, body mass index (BMI), and blood pressure were collected. Biochemical parameters comprised serum electrolytes, lipid profile, liver and kidney function, direct renin concentration (DRC), plasma aldosterone concentration (PAC), angiotensin II (Ang II), plasma cortisol rhythm, and adrenocorticotropic hormone (ACTH) (measured via UniCel DxI 600 Access, Beckman, USA). PAC and DRC were quantified by the Soling chemiluminescence assay kit and analyzer (Liaison XL, China).

#### 2.2.2 Echocardiographic and Imaging Examination

Left ventricular end-diastolic diameter (LVEDD), left ventricular posterior wall thickness in diastole (LVPWT), interventricular septal thickness (IVST), relative wall thickness (RWT), and left atrial diameter (LAD) were measured in the parasternal long-axis view. Left ventricular ejection fraction (LVEF) was determined by the Biplane Simpson's method. In the four-chamber apical view, pulsed-wave Doppler measured peak velocities of early (E) and late (A) atrial inflow 13 to evaluate the impact of LV relaxation on mitral E velocity. Early diastolic velocity (e' velocity) was measured, and the E/e' ratio was computed. Established indicators for LV diastolic dysfunction include e' velocity, the E/A ratio, and E/e' ratio 13, 14 (Supplementary Material). Abdominal computed tomography (CT) categorized adrenal nodules into three types. (1) unilateral lesion (unilateral adrenal nodule ≥10 mm or thickening, without contralateral thickening); (2) bilateral lesions (bilateral adrenal nodules ≥10 mm or thickening); or (3) bilateral non-obvious lesions (bilateral adrenal nodules <10 mm or without thickening).

#### 2.2.3 Visceral Fat Deposition Measurement

**Figure 2:**
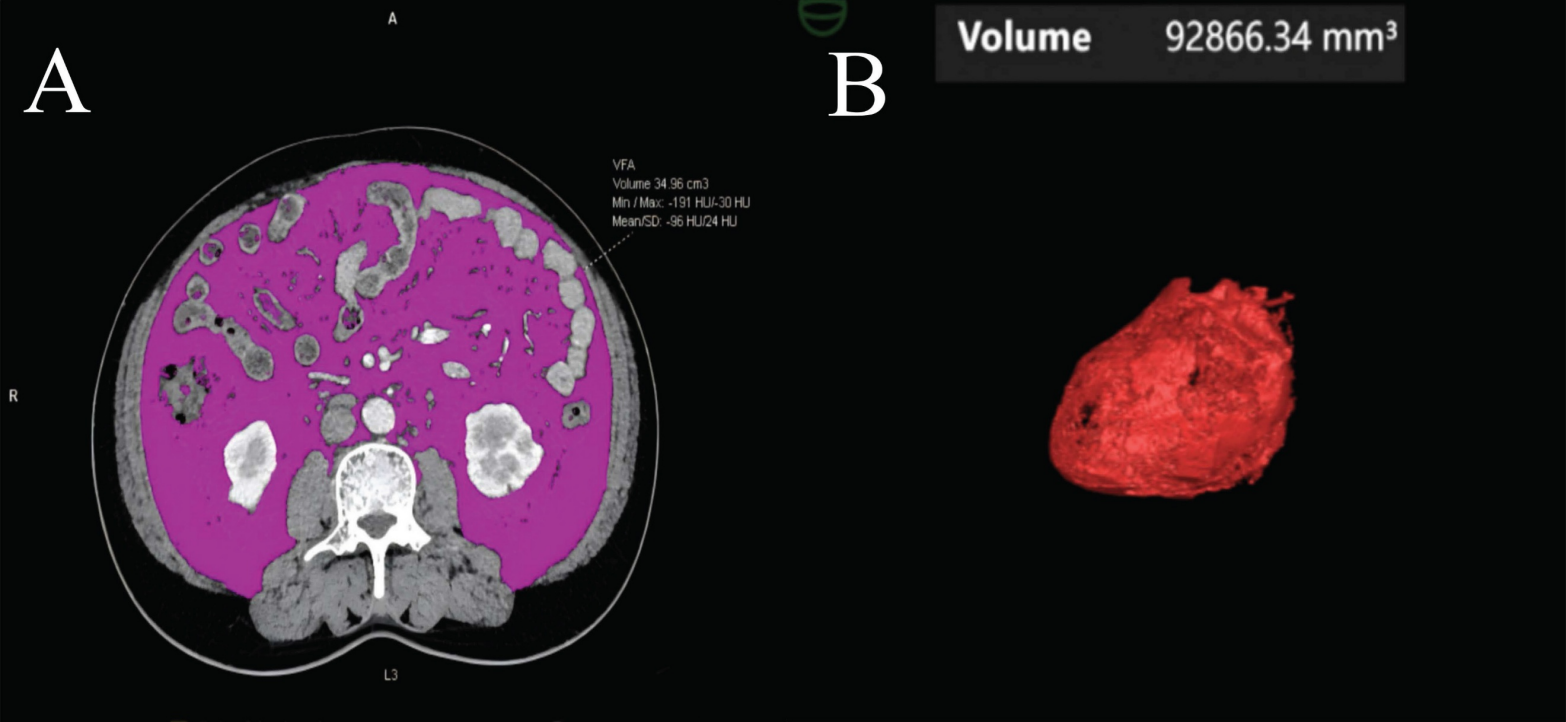
** Visceral fat and epicardial adipose tissue volume measurements from a CT scan. (A)** The visceral fat area (VFA) was measured at the third lumbar vertebra. Within this region of interest, pixels between -190 and -30 Hounsfield Units were accounted as fat. **(B)** After 3-dimensional reconstruction, EAT volume was calculated by summation of all pixels accounted as fat.

Abdominal CT images obtained during the portal venous phase at diagnosis were analyzed using body composition analysis software (Syngo.via Client 10.6, Germany). The software automatically detects the third lumbar vertebra (L3) and measures visceral fat area (VFA) (Figure 2A), subcutaneous fat area (SFA), skeletal muscle area (SMA), and intermuscular fat area (IMFA) at the L3 level 15-17. Renal sinus fat (RSF) 18, 19, hepatic steatosis, and epicardial adipose tissue (EAT) volume 20-23 were also quantified (Figure 2B) (Supplementary Material).

### 2.3 Overview of the Analytical Workflow

The analytical workflow comprised four phases: (1) data imputation and partitioning; (2) Feature Selection and Class Imbalance; and (3) Model Development and Evaluation. Continuous variables were assessed for normality (Kolmogorov-Smirnov or Shapiro-Wilk test) and compared using Student's t-test (presented as mean ± SD) or the Mann-Whitney U test (median IQR). Categorical variables were compared via chi-square tests. These analyses evaluated baseline clinical and abdominal fat differences between lateralization groups across sexes. Analyses were performed using SPSS 27.0 and R 4.4.3. Statistical significance was set at two-tailed P < 0.05.

#### 2.3.1 Data Preprocessing and Partitioning

Missing data were addressed via multiple imputation (predictive mean matching for continuous variables; logistic regression for binary). To prevent data leakage, the cohort was randomly split into training (70%) and testing (30%) sets; all preprocessing was strictly restricted to the training set.

#### 2.3.2 Feature Selection and Class Imbalance

LASSO regression identified robust UPA predictors, retaining features with non-zero coefficients based on the optimal penalty parameter (λ.min) derived from 10-fold cross-validation. To mitigate class imbalance without compromising validation integrity, the Synthetic Minority Over-sampling Technique (SMOTE) (via the themis R package) generated synthetic samples exclusively within each training fold during cross-validation.

#### 2.3.3 Model Development and Evaluation

Five ML algorithms, including logistic regression, extreme gradient boosting (XGBoost), support vector machine (SVM), Naive Bayes, and Random Forest (RF), were trained using 10-fold stratified cross-validation for hyperparameter optimization. Model performance was evaluated using receiver operating characteristic (ROC) curve analysis, area under the curve (AUC) values, accuracy, recall, and F1 score, which were calculated for each imputation and averaged across the 10 datasets. Finally. Shapley additive explanations (SHAP) analysis was used to interpret model predictions by quantifying the contribution of each feature. Global interpretation summarized overall feature importance across the population, whereas local interpretation explained feature-specific contributions for individual predictions.

## 3. Results

### 3.1 Comparison of Clinical Biochemical Characteristics and Body Composition in BPA and UPA Groups (Figure 3)

A total of 122 BPA and 113 UPA patients were enrolled in the study. The results showed systolic blood pressure (SBP) was higher, and serum potassium and renin levels were significantly lower in the UPA compared to the BPA group (P < 0.05). Additionally, PAC and ARR were higher in the UPA group both at baseline and after saline infusion (P < 0.001). In terms of body composition, the BPA group showed greater VFA (113.12 [76.53, 164.00] vs. 102.55 [63.77, 134.03] cm², P = 0.049) and SFA (123.60 [105.80, 155.68] vs. 103.15 [85.38, 148.51] cm², P = 0.027) compared to the UPA group. Conversely, the UPA group had a larger maximum nodule diameter (13 10, 16 vs. 11 8, 15 mm, P = 0.017) and more patients presenting nodules ≥ 10 mm (72 [63.7%] vs. 54 [44.3%], P=0.002). Moreover, the UPA group was associated with a higher LI (P < 0.001) and lower CI, while the BPA group demonstrated higher atrial filling velocity (P = 0.021). There were no significant differences in cardiac function, cardiac fat deposition, RSF volume, or hepatic steatosis between the two groups. In summary, the UPA group presented with prominent hypertension and hypokalemia, whereas the BPA group showed more visceral fat deposition.

### 3.2 Sex-Specific Differences in Clinical Biochemical Characteristics of BPA and UPA Groups (Table 1)

The BPA and UPA groups were further stratified by sex, with 111 males (60 with BPA, 51 with UPA) and 124 females (62 with BPA, 62 with UPA). In males, the BPA group exhibited higher BMI and body weight than the UPA group, whereas no differences were found in females. In contrast, the female BPA group showed higher triglycerides (TG) (1.62 [1.19, 3.20] vs. 1.23 [0.77, 1.83] mmol/L, P = 0.007), higher serum calcium (P = 0.044), and lower serum sodium (P = 0.01) compared to UPA. Additionally, males in both subtypes exhibited higher creatinine and uric acid compared to females (P < 0.001). These findings suggest that the clinical biochemical characteristics of PA patients exhibited sex and subtype-specific patterns, with the male BPA group demonstrating elevated BMI and body weight, while the female BPA group showed dyslipidemia and electrolyte disturbances.

### 3.3 Sex-Specific Adipose Tissue Deposition in BPA and UPA Groups (Figure 3)

The BPA group displayed markedly higher VFA/SFA compared to the UPA group (P = 0.001), with VFA/BMI remaining significantly different after BMI adjustment (6.50±2.19 vs. 4.88±1.99 cm²·kg⁻¹·m⁻², P = 0.002). Notably, the male BPA group exhibited more pronounced RSF volume deposition than the UPA group (2.78 [1.73, 4.08] vs. 1.82 [1.12, 3.85] cm³, P = 0.049), particularly in the right kidney, but disappeared after adjusting for renal volume. Across both subtypes, males demonstrated more pronounced ectopic deposition of visceral, subcutaneous, and renal sinus fat compared to females (P < 0.001). Furthermore, the male BPA group had significantly larger EAT volume than the UPA group (144.69 [103.65, 167.56] vs. 97.75 [91.91, 123.82] cm³, P = 0.038), which was associated with altered cardiac diastolic function, including increased atrial filling velocity and E/e'. In the BPA group, males exhibited more significant cardiac fat deposition than females. In contrast, females showed no significant subtype differences in abdominal fat, cardiac fat, or cardiac function parameters. These findings indicate that the male BPA group demonstrated pronounced ectopic fat accumulation in the abdominal and epicardial compartments alongside altered cardiac diastolic function, whereas no subtype differences were observed in the female BPA group.

### 3.4 Sex-Specific Adrenal Nodule Characteristics of BPA and UPA Group (Table 1)

In females, the UPA group displayed larger maximum nodule diameter compared to the BPA group, with more patients having nodules with a diameter ≥ 10 mm (42 [69.4%] vs. 23 [37.1%], P = 0.001). No significant differences in CT nodule characteristics were discovered between subtypes in males.

### 3.5 Prediction Models for PA Subtyping in Different Sexes

**Figure 4:**
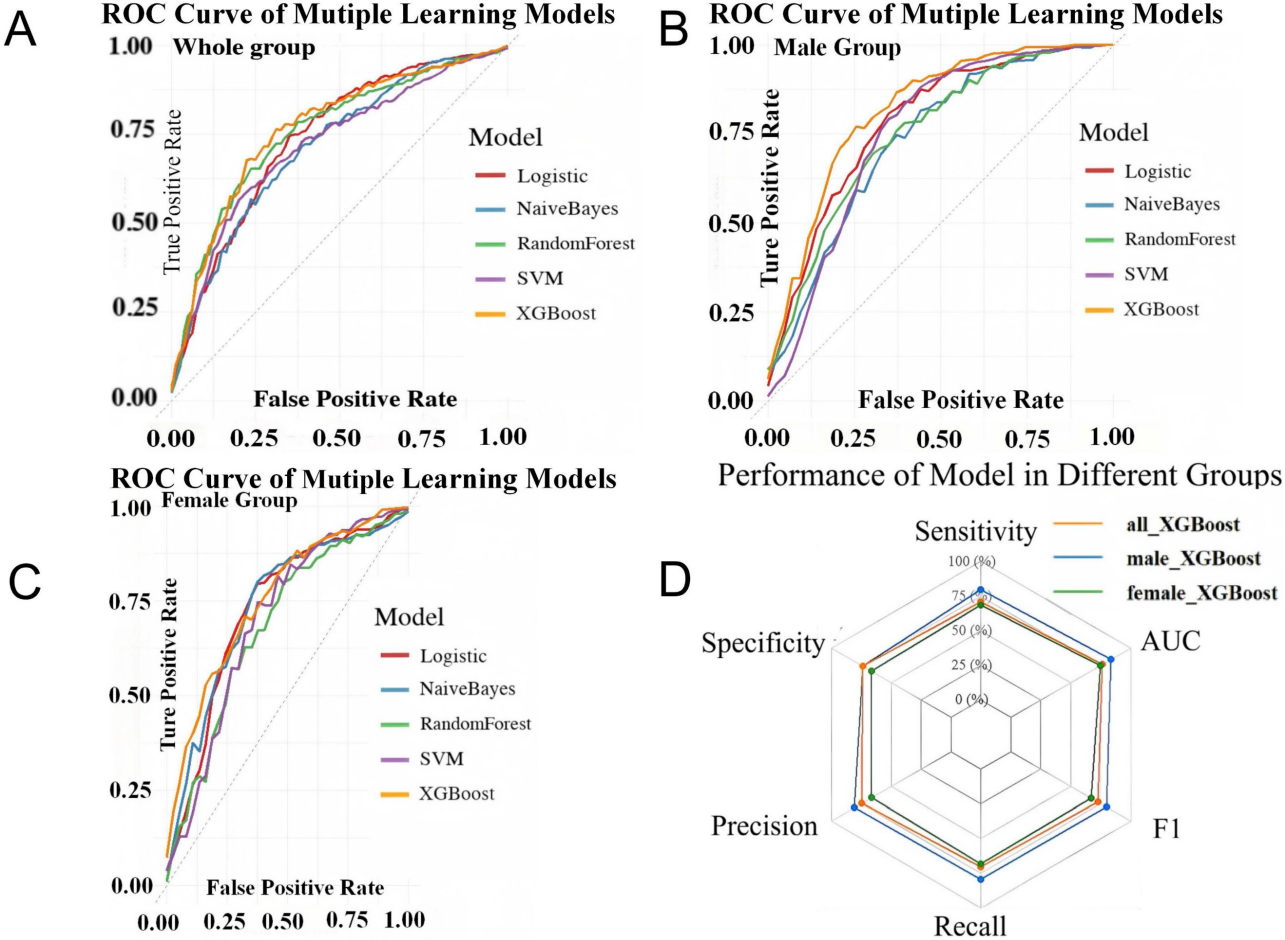
** Validation set performance for the models across each patient cohort.** ROC Curves Comparison of Multiple Machine Learning Models in Whole group **(A)**, male group **(B)** and female group **(C)**. Performance of XGBoost model in different groups **(D)**. LASSO: least absolute shrinkage and selection operator; XGBoost: extreme gradient boosting; SVM: support vector machine; ROC: receiver operating characteristic curve.

Sex-stratified XGBoost models were developed after LASSO feature selection. The male model integrated VFA, SBP, the lowest serum potassium during hospitalization (K), and aldosterone levels, whereas the female model incorporated TG, K, PAC after SIT, and CT lesion characteristics. The male model achieved superior discriminative performance (AUC 0.84 ± 0.05) compared with both the female (AUC 0.75 ± 0.02) and overall models (AUC 0.77 ± 0.02), following 10-fold cross-validation with multiple imputation (Figure 4).

**Figure 5:**
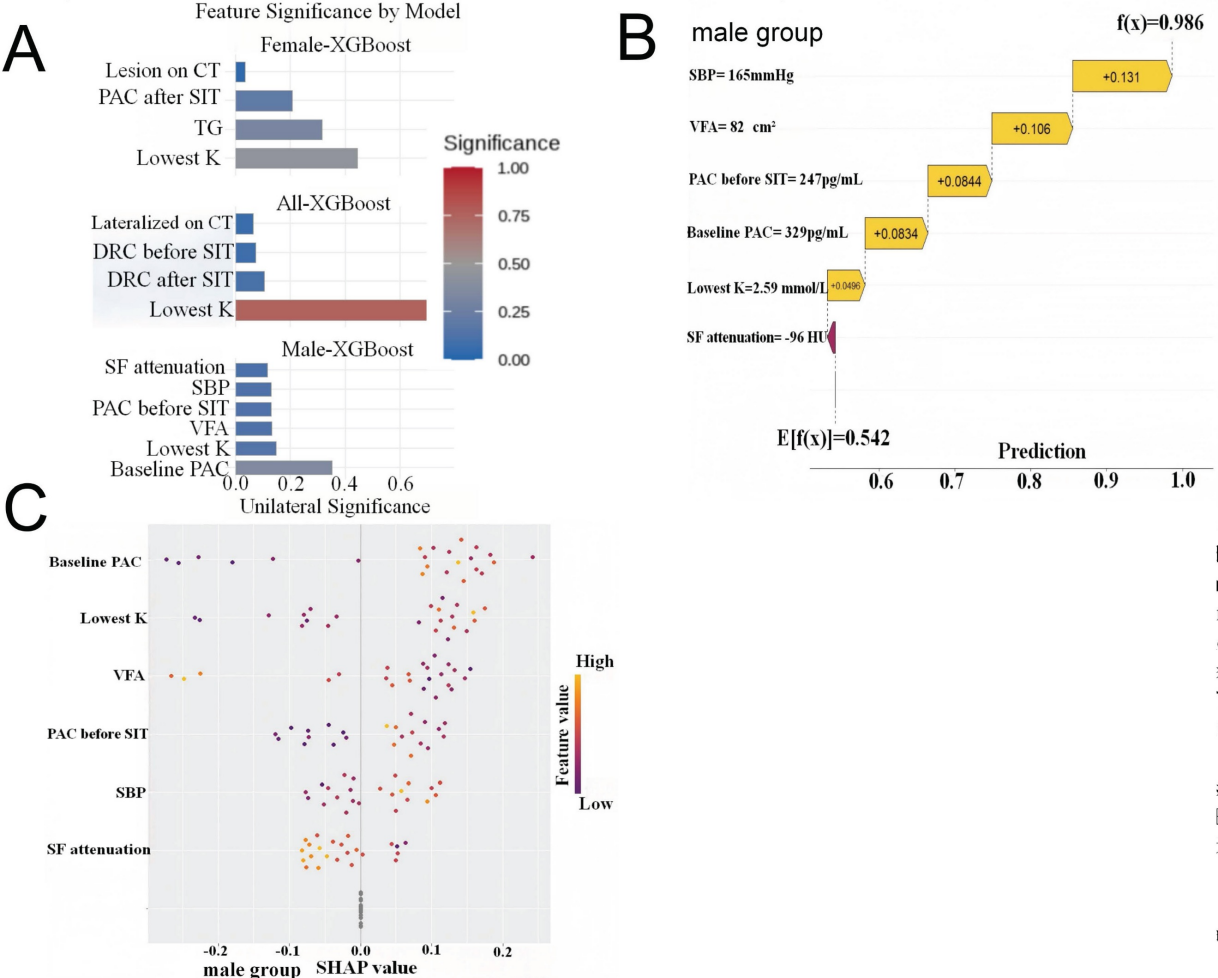
** Waterfall Plots of SHAP Values Exploring the Important Expectation. (A)** Features ranked by their mean absolute SHAP values in different primary aldosteronism (PA) groups. The extracted feature after LASSO regression for primary aldosteronism of 1 sample in the male group **(B)**. SBP and VFA played important predictive roles in the male group, the SBP=165mmHg increased the prediction probability by 0.131, while VFA=82cm^2^ increased it by 0.106. The final model output indicated a probability of 0.986 for the “unilateral group”. **(C)** The extracted feature after LASSO regression for primary aldosteronism of all samples in the male group. SHAP: shapley additive explanations; LASSO: least absolute shrinkage and selection operator; SBP: systolic blood pressure; VFA: visceral fat area; Lowest K: the lowest potassium during hospitalization; DRC: direct renin concentration; SIT: saline infusion test; CT: computed tomography; PAC: plasma aldosterone concentration; TG: triglycerides; SF: subcutaneous fat.

SHAP analysis revealed distinct pathophysiological drivers of lateralization between sexes (Figure 5). In males, visceral fat reduction emerged as a critical positive predictor of UPA, with a VFA of 82 cm² and SBP of 165 mmHg substantially increasing prediction probabilities (SHAP contributions +0.106 and +0.131, respectively). K and baseline PAC demonstrated consistent directional contributions across the male cohort. Conversely, UPA prediction in females was primarily driven by CT lesion features, TG levels, and PAC after SIT values. These divergent patterns suggest that lateralization in males is metabolically driven, while in females, biochemical features play a dominant role, indicating distinct mechanistic pathways between sexes.

## 4. Discussion

### 4.1 UPA Exhibits More Significant Clinical Features, While BPA Shows Greater Visceral Fat Accumulation

This study confirmed that the UPA group presented with higher blood pressure, lower serum potassium, and more elevated PAC and ARR, consistent with previous research 9, 24, 25. Conversely, the BPA group was characterized by obesity and abdominal fat accumulation, particularly visceral fat accumulation, accompanied by higher BMI and waist circumference 5, 10, 25-27. We further demonstrated more pronounced abdominal fat deposition compared to cardiac fat in BPA. Subtype-specific patterns of aldosterone and adiposity were noted. ARR negatively correlates with visceral fat deposition parameters (VFA, waist circumference, and BMI) in UPA 27, whereas PAC has a positive correlation with VFA and visceral fat percentage (VF%) in BPA 28. These differences suggest distinct metabolic phenotypes between subtypes. The potential mechanism involves excessively high local PAC concentration in UPA, inhibiting adipocyte differentiation and activating mineralocorticoid receptor (MR)-mediated inflammation and fibrosis 27, which leads to adipocyte atrophy. Conversely, BPA is typically associated with elevated systemic PAC levels, where visceral fat stimulates the renin-angiotensin-aldosterone system (RAAS) and inflammatory factors to exacerbate ectopic fat accumulation. Additionally, genetic heterogeneity, such as KCNJ5 mutation status, may partially explain variability in metabolic profiles across subtypes 29. But our data primarily support a clinical association rather than direct mechanistic inference.

### 4.2 Metabolic Characteristics of PA Subtypes Exhibit Sex Differences, With Male BPA Showing Higher Cardiovascular Risk Than UPA

We observed significant sex-specific differences in PA subtypes. Male group with BPA had significantly higher body weight, visceral, renal sinus, and epicardial fat deposition than UPA, accompanied by cardiac diastolic dysfunction and higher VFA/BMI ratio, suggesting increased cardiovascular risk. In contrast, no significant differences in visceral fat were observed between subtypes in females, although higher triglyceride levels were noted in female BPA. Previous research has found that male PA patients in two subtypes exhibited more elevated markers of early renal impairment (serum creatinine, 24-hour urinary microalbumin) and higher cardiovascular risk than females 7, similar to our findings. Nevertheless, previous studies indicated a greater metabolic risk in female BPA patients, as opposed to female UPA 5, 6, 9. Our study demonstrated more severe metabolic disturbances in the male BPA group with more fat accumulation. We speculate that the sex difference arises from (1) cyclical fluctuations of female sex hormones and oral contraceptives, affecting RAAS and ARR variability 30. (2) KCNJ5 mutations being more common in female UPA 31, while mutations in CACNA1D and ATP1A1 are more prevalent in males 32, 33. (3) Hormonal level differences: estrogen exerts cardioprotective effects, thereby counteracting PAC-induced target organ damage 7. Studies have suggested that testosterone may downregulate aldosterone synthase expression, thereby reducing PAC levels 34. Hormonal effects on fat distribution, with estrogen promoting subcutaneous fat storage and androgens promoting visceral fat accumulation 35. Furthermore, visceral fat in both sexes secretes inflammatory factors that exacerbate metabolic disturbances 36, 37. Prospective studies are needed for sex-specific mechanisms in PA fat distribution.

Recent research has found that perirenal fat thickness is significantly positively related to hypertension risk in BPA, with a stronger association in obese individuals 38. We speculate that sex hormones may influence renal sinus fat accumulation independently of PAC levels due to tissue-specific receptor distribution 39, 40. Additionally, we have found that patients with BPA had a higher risk of cardiac diastolic dysfunction, particularly pronounced in males, potentially related to epicardial fat deposition. Prospective stratified studies are needed for validation.

### 4.3 Construction of Sex-Specific PA Prediction Models

#### 4.3.1 Clinical Significance

PA subtyping is crucial in clinical practice. Biochemical indicators, including renin activity, aldosterone cutoff concentrations 41, 42, and urinary steroid profiles 43, 44, served as screening tools for PA subtypes. Although biochemical markers and imaging are widely used, their diagnostic thresholds vary across centers, and CT has limited sensitivity for small lesions (< 1 cm) or bilateral disease. While AVS remains the gold standard for lateralization 11, 45, its invasiveness and technical complexity limit widespread use. Our study identified sex-specific differences in metabolic and imaging features, contributing to the development of a multimodal prediction model. By integrating clinical, biochemical, and visceral fat variables, our approach provides a more comprehensive framework for non-invasive subtype prediction.

#### 4.3.2 Model Interpretability

We used XGBoost to capture non-linear relationships among variables, enhancing predictive accuracy over conventional models. Sex-stratified modeling improved discrimination and revealed distinct feature patterns. In males, VFA, K, SBP, and aldosterone were key predictors, with VFA contributing substantially to model performance. The female model incorporated TG, K, PAC after SIT, and CT nodule characteristics. Besides, SHAP analysis improved interpretability by quantifying feature contributions at both group and individual levels. VFA was among the most influential predictors in males, whereas biochemical features predominated in females, suggesting sex-specific diagnostic pathways. And model stability was confirmed by cross-validation.

#### 4.3.3 Clinical Application

Our model allows for pre-selection of surgical candidates through quantitative UPA probability scoring. By integrating metabolic and biochemical features beyond imaging, our models may expand the AVS-sparing surgical candidate pool with better risk stratification. And SHAP-based explanations enable personalized predictions and clearer communication at the individual level.

### 4.4 Advantages and Limitations

The PA subtype prediction model developed in this study has several advantages (1) Integration of abdominal fat parameters with biochemical and clinical features; (2) Semi-automated fat measurement software facilitates convenient data acquisition, avoiding challenges such as standardizing radiomics features; (3) Sex-stratified modeling aligns with clinical individualized and precision treatment strategies; (4) Subtyping was performed using AVS as the gold standard, ensuring reliability.

This study still has several limitations: first, the retrospective design resulted in missing data; despite multiple imputation, selection bias remains a potential risk. Second, while the model can predict subtypes, it does not determine the dominant side of PA. Further prospective studies are required to validate the model's generalizability.

In summary, this study found that UPA presented with more typical clinical phenotypes, whereas BPA was associated with higher cardiovascular risk factors, particularly in males, where it was correlated with more severe ectopic fat accumulation in abdominal, renal, and cardiac regions. The two PA subtypes exhibit significant sex differences. Thus, sex-specific prediction models developed may provide innovative tools for clinical precision subtyping and prognostic assessment.

## Supplementary Material

Supplementary methods.

## Figures and Tables

**Figure 3:**
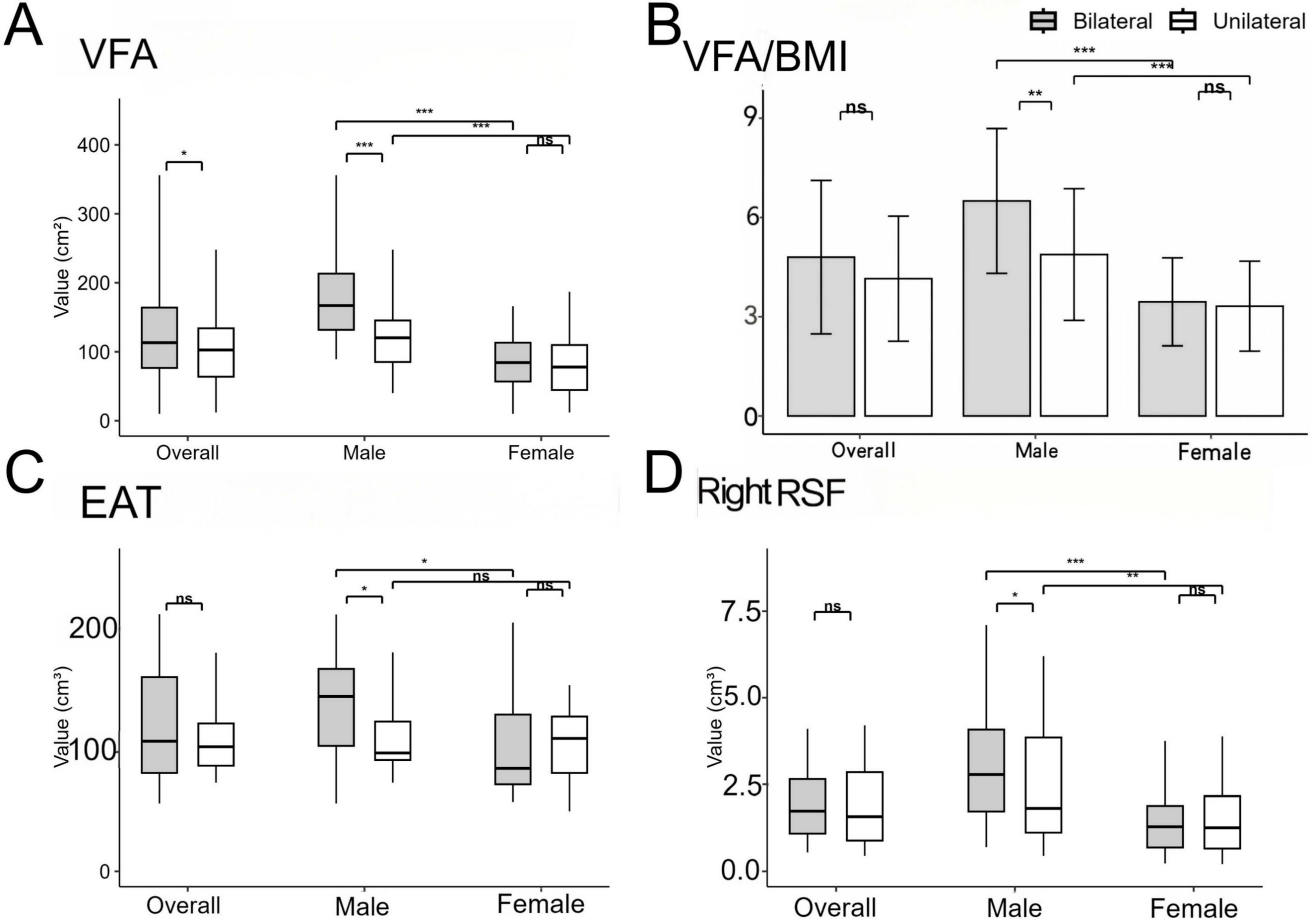
** Comparison of body composition of PA patients according to subtype and sex.** Comparison of VFA **(A)**, VFA/BMI **(B)**, EAT **(C)**, right RSF **(D)** between unilateral and bilateral PA patients for each sex. *P < 0.05, **P < 0.01, and ***P < 0.001. P values were calculated using Mann-Whiteny test or Mann-Whitney U test. PA: primary aldosteronism; VFA: visceral fat area; BMI: body mass index; EAT: epicardial adipose tissue; RSF: renal sinus fat.

**Table 1:** Clinical biochemical characteristics of PA patients according to sex and subtype.

Variables	Male			Female		
	Unilateral PA (n = 51)	Bilateral PA (n = 60)	P	Unilateral PA (n = 62)	Bilateral PA (n = 62)	P
Age (years)	50 [39,61]^a^	51 [40,57]^a^	0.65	49 [40,59]^a^	52 [44,61]^a^	0.498
BMI (kg/m2 )	25.41 ±2.98^b^	26.68±2.89^a^	0.05	24.58±4.27^b^	24.29±2.87^b^	0.73
SBP (mmHg)	144 [134,156]^a^	139 [129,145]^b^	0.012	140 [130,148]^a^	134 [128,146^]ab^	0.268
DBP (mmHg)	91 [85,102]^a^	90 [83,95]^a^	0.271	85 [81,94]^b^	86 [80,93]^ab^	0.779
Lowest K (mmol/L)	3.20 [2.77,3.50]^b^	3.62 [3.27,3.89]^a^	0.001	3.16 [2.70,3.57]^b^	3.57 [3.45,3.82]^a^	0.001
Na (mmol/L)	142.15 ±2.26^a^	141.51±2.02^ab^	0.175	142.01±2.79^a^	140.55 ±2.08^b^	0.01
Cl (mmol/L)	102.27±3.45^b^	103.69 ±2.83^a^	0.042	103.85±3.51^b^	103.61 ±2.76^ab^	0.733
Ca (mmol/L)	2.31 [2.27, 2.36]^ ab^	2.31 [2.24,2.36]^a^	0.919	2.25 [2.19,2.35]^b^	2.31 [2.26,2.37]^a^	0.044
TG (mmol/L)	2.43 [1.61,3.04]^a^	2.21 [1.25,4.24]^a^	0.853	1.23 [0.77,1.83]^b^	1.62 [1.19,3.20]^a^	0.007
eGFR(ml/min/1.73^2)	83.70 [67.73,99.30]^b^	87.50 [68.45,102.05]^ab^	0.738	96.60 [85.45 ,111.75]^a^	96.90 [80.30,105.90]^a^	0.53
Creatinine (umol/L)	92.50 [79.25,107.25]^a^	92.00 [76.50,102.50]^a^	0.725	65.00 [56.00,72.50]^b^	63.00 [55.50,75.00]^b^	0.728
uric acid (umol/L)	383.12±92.21^a^	407.61 ±100.07^a^	0.244	303.89±100.72^b^	283.10 ±70.47^b^	0.304
PAC (pg/mL)	307.00 [229.25,422.75]^a^	196.50 [155.25,337.50]^b^	0.013	282.50 [192.25,382.75]^a^	207.00 [127.00 ,277.50]^b^	0.01
ARR	121.25 [77.52,297.25]^a^	70.00 [28.89,145.65]^b^	0.01	158.78 [102.73,344.83]^a^	61.20 [35.15 ,130.37]^b^	0.001
PAC before SIT (pg/mL)	281.00 [242.50,385.75]^a^	181.00 [130.00,305.00]^b^	0.008	264.00 [193.00,458.50]^a^	188 [104.80 ,223.00]^b^	0.002
ARR before SIT	141.47 [70.32,251.35]^a^	108.18 [37.98,204.58]^ab^	0.291	191.82 [151.88,432.00]^a^	80.00 [43.10 ,161.70]^b^	0.006
PAC reduction rate	0.32 [0.15,0.52]^ab^	0.34 [0.09,0.48]^a^	0.665	0.20 [0.02 ,0.49]^b^	0.41 [0.32 ,0.61 ]^a^	0.019
PAC after SIT (pg/mL)	175.00 [123.00,283.25]^a^	110.00 [88.30,205.00]^b^	0.039	197.0 [133.5,326.0]^a^	80.7 [62.7,128.0]^b^	0.001
ARR after SIT	119.64 [91.38,302.92]^a^	57.90 [45.90,117.46]^b^	0.011	113.92 [58.32 ,240.31]^a^	65.78 [29.22 ,131.56]^ab^	0.099
LI	19.96 [9.18,46.21]^a^	1.92 [1.40,2.64]^c^	0.001	13.65 [4.89,30.15]^b^	1.71 [1.18,2.32]^c^	0.001
CI	0.17 [0.09,0.38]^c^	0.65 [0.22,1.38]^b^	0.001	0.29 [0.13,0.56]^c^	1.21 [0.62,1.69]^a^	0.001
Duration of HTN (years)	6 2,10^a^	7.00 [3.00,10.50]^a^	0.521	5.5 [2.25,13.75]^a^	6 3,10^a^	0.853
HTN, n(%)	44(86.3%)^b^	58(96.7%)^a^	0.046	55(88.7%)^b^	51(82.3%)^b^	0.308
Lesion on CT excess 10 mm, n(%)	30(58.8%)^a^	31(51.7%)^ab^	0.450	42(69.4%)^a^	23(37.1%)^b^	0.001
Lateralized on CT, n(%)	30(58.8%)^a^	19(31.7%)^b^	0.004	40(64.5%)^a^	14(22.6%)^b^	0.001
Lesson diameter (mm)	12.50 [9.25,15]^a^	11.00 8,15^ab^	0.362	14.00 [11.00 ,17.00]^a^	10 [8,14.25]^b^	0.011

Student t test and Chi-square test were carried out between different sexes in the same subtype PA group, which were also carried out between different subtype PA groups in the same sexes. Continuous data are presented as mean (standard deviation), median (interquartile range) based on normality, and categorical variables are expressed as number (%). Values within the same row with different lowercase letters are significantly different (P < 0.05). PA: primary aldosteronism; BMI: body mass index; SBP: systolic blood pressure; DBP: diastolic blood pressure; K: potassium; Na: sodium; Cl: serum chloride; Ca: calcium; TC: total cholesterol; TG: triglycerides; HDL-C: high-density lipoprotein cholesterol; LDL-C: low-density lipoprotein cholesterol; eGFR: estimated glomerular filtration rate; PAC: plasma aldosterone concentration; ARR: aldosterone renin ratio; DRC: direct renin concentration; SIT: saline infusion test; LI: (lateralization index); SI: (Selectivity Index); CI: (central index); HTN: hypertension; CT: computed tomography.

## Data Availability

In accordance with the Declaration of Helsinki, this study was approved by the Medical Research Ethics Committee of Tongji Hospital, Tongji Medical College, Huazhong University of Science and Technology (approval No.#TJ-IRB202407043). All the original data presented in the study was in the article or supplementary material. Further inquiries can be made to the corresponding author reasonably.

## Abbreviations

PA: primary aldosteronism
UPA: unilateral PA
BPA: bilateral PA
VFA: visceral fat area
SFA: subcutaneous fat area
V/S: visceral fat area/subcutaneous fat area
BMI: body mass index
EAT: epicardial adipose tissue
RSF: renal sinus fat
HU: Hounsfield unit
SHAP: Shapley additive explanations
SBP: systolic blood pressure
Lowest K: the lowest potassium during hospitalization
DRC: direct renin concentration
SIT: saline infusion test
CT: computed tomography
PAC: plasma aldosterone concentration
TG: triglycerides
AVS: adrenal venous sampling
CCT: captopril challenge test
SIT: saline infusion test
SI: selectivity index
LI: lateralization index
CI: central index
Ang II: angiotensin II
ACTH: adrenocorticotropic hormone
LVEDD: Left ventricular end-diastolic diameter
LVPWT: left ventricular posterior wall thickness in diastole
IVST: interventricular septal thickness
RWT: relative wall thickness
LAD: left atrial diameter
LVEF: left ventricular ejection fraction
RAAS: renin-angiotensin-aldosterone system
LASSO: least absolute shrinkage and selection operator
CV: cross-validation
XGBoost: extreme gradient boosting
SVM: support vector machine
RF: random forest
ROC: receiver operating characteristic curve
