# Supplementary material for: The diagnostic role of machine learning models related to visceral fat with sex-specific distribution in subtypes of primary aldosteronism
Source: Int J Med Sci. 2026 Apr 23;23(6):1982–91. doi: 10.7150/ijms.131815 (PMC13181375; doi:10.7150/ijms.131815)
Supplement: Supplementary file 1 — Supplementary methods. [file ijmsv23p1982s1.pdf]

Supplementary Methods

1. Confirmatory Tests

The captopril challenge test was considered positive if aldosterone suppression rate was <30% or aldosterone level ≥11 ng/dL at 2 hours after oral administration of 50 mg captopril. The saline infusion test was positive if aldosterone level >10 ng/dL after 4-hour intravenous infusion of 2 L 0.9% sodium chloride solution; levels of 5 – 10 ng/dL were considered borderline positive.

2. Adrenal Venous Sampling (AVS)

The selectivity index (SI) was calculated as the ratio of cortisol concentration in the adrenal vein to that in the inferior vena cava to confirm adequate cannulation, with SI ≥2:1 (without ACTH stimulation) or ≥3:1 (with ACTH stimulation) indicating successful sampling. The lateralization index (LI) was calculated as the aldosterone/cortisol ratio (ACR) of the higher side divided by the contralateral ACR. The contralateral suppression index (CI) was calculated as the ACR of the lower side divided by the ACR of the distal inferior vena cava. Unilateral PA was defined as LI ≥4 post-ACTH stimulation or LI ≥2 without ACTH stimulation.

3.Echocardiography examination

All subjects underwent transthoracic echocardiography performed by professional sonographers using a GE Vivid E9 ultrasound system (GE Vingmed Ultrasound, Horten, Norway) equipped with a 1.7 – 3.3 MHz transducer, following American Society of Echocardiography (ASE) guidelines[1]. In the parasternal long-axis view, left ventricular end-diastolic diameter (LVEDD), interventricular septal thickness (IVST), left ventricular posterior wall thickness in diastole (LVPWT), and left atrial diameter (LAD) were measured. Left ventricular ejection fraction (LVEF) was assessed using the biplane Simpson's method. Pulsed-wave Doppler in the apical four-chamber view was used to measure peak early (E) and late (A) transmitral inflow velocities. The E/A ratio was calculated as an index of left ventricular diastolic function; an E/A ratio <1 indicated impaired myocardial relaxation[2]. Mitral annular early diastolic velocity (e' velocity) and the ratio of early transmitral flow velocity to mitral annular velocity (E/e') were also measured. The E/A ratio, e' velocity, and E/e' ratio are known as indices for LV diastolic dysfunction[3].

4. Imaging examination

Abdominal computed tomography (CT) scans were performed on a 64-slice CT scanner using the following parameters: 120 kVp, 0.5-second rotation time, and 1.25 mm slice thickness. Portal venous phase contrast-enhanced abdominal CT images obtained at diagnosis were analyzed by investigators blinded to clinical diagnoses using body composition analysis software (Syngo.via Client 10.6, Siemens, Germany). The software automatically detected the third lumbar vertebra

(L3) and measured skeletal muscle area (SMA), visceral fat area (VFA), subcutaneous fat area (SFA), and intermuscular fat area (IMFA) at the L3 level. Mean CT attenuation values (Hounsfield units, HU) were recorded for each parameter (Figure 2). To compare body composition across groups, SMA, VFA, SFA, and IMFA were normalized to body mass index (BMI, SMA/BMI, VFA/BMI, SFA/BMI, IMFA/BMI)[4–6].

(1) Renal sinus fat (RSF) Measurement

RSF boundaries were defined by visual inspection as the linear trajectory extending from the indentation between two adjacent poles across the renal sinus opening, thereby excluding surrounding abdominal adipose tissue from the measurement (Figure 2). To adjust for kidney size, the ratio of RSF tissue volume to corresponding total kidney volume was calculated using the arithmetic mean for each subject[7,8].

(2) Epicardial Adipose Tissue (EAT) Measurement

Cardiac fat deposition was quantified using TIMESLicepro (https://slice-doc.netlify.app/;TIMESLicepro v5.0.2; China). EAT volume was defined from the level of pulmonary artery bifurcation to pericardial disappearance, with the EAT region identified as fat tissue between the myocardium and pericardium. Adipose tissue was defined by CT attenuation values between -190 and -30 HU[9–12].

(3) Hepatic Steatosis Measurement

Two circular regions of interest (ROI) of 1.5 cm^2 were placed at two different sites in each segment of the right hepatic lobe (segments V, VI, VII, and VIII according to the Couinaud system). Hepatic attenuation (L) was calculated as the mean HU value across all eight ROIs. Spleen attenuation (S) was obtained by averaging HU values from three 1.5 cm^2 circular ROIs placed in the upper, middle, and lower thirds of the spleen. Portal vein (P) and abdominal aorta (A) attenuation were measured by placing three circular ROIs as large as possible in the main portal trunk and aorta at the celiac axis level across three different images, respectively. Hepatic steatosis was calculated using the formulas: $L\text{-}B = [L - 0.3 \times (0.75 \times P + 0.25 \times A)] / 0.7$, L-S, and L/S ratio (Figure 2)[13,14].

Reference

1. Lang RM, Badano LP, Mor-Avi V, et al. Recommendations for cardiac chamber quantification

by echocardiography in adults: an update from the american society of echocardiography and the european association of cardiovascular imaging. *Eur Heart J, Cardiovasc Imaging*. 2015;16(3):233-270. doi:10.1093/ehjci/jev014

2. Oh JK, Hatle L, Tajik AJ, Little WC. Diastolic heart failure can be diagnosed by comprehensive two-dimensional and doppler echocardiography. *J Am Coll Cardiol*. 2006;47(3):500-506. doi:10.1016/j.jacc.2005.09.032

3. Nagueh SF, Smiseth OA, Appleton CP, et al. Recommendations for the Evaluation of Left Ventricular Diastolic Function by Echocardiography: An Update from the American Society of Echocardiography and the European Association of Cardiovascular Imaging. *J Am Soc Echocardiogr*. 2016;29(4):277-314. doi:10.1016/j.echo.2016.01.011

4. Kays Mohammed Ali Y, Dolin TG, Damm Nybing J, et al. Change in abdominal obesity after colon cancer surgery - effects of left-sided and right-sided colonic resection. *Int J Obes (2005)*. 2024;48(4):533-541. doi:10.1038/s41366-023-01445-8

5. Wajchenberg BL. Subcutaneous and visceral adipose tissue: their relation to the metabolic syndrome. *Endocr Rev*. 2000;21(6):697-738. doi:10.1210/edrv.21.6.0415

6. Tolonen A, Pakarinen T, Sassi A, et al. Methodology, clinical applications, and future directions of body composition analysis using computed tomography (CT) images: A review. *Eur J Radiol*. 2021;145:109943. doi:10.1016/j.ejrad.2021.109943

7. Krievina G, Tretjakovs P, Skuja I, et al. Ectopic Adipose Tissue Storage in the Left and the Right Renal Sinus is Asymmetric and Associated With Serum Kidney Injury Molecule-1 and Fibroblast Growth Factor-21 Levels Increase. *EBioMedicine*. 2016;13:274-283. doi:10.1016/j.ebiom.2016.10.020

8. Wagner R, Machann J, Lehmann R, et al. Exercise-induced albuminuria is associated with perivascular renal sinus fat in individuals at increased risk of type 2 diabetes. *Diabetologia*. 2012;55(7):2054-2058. doi:10.1007/s00125-012-2551-z

9. Nakazato R, Shmilovich H, Tamarappoo BK, et al. Interscan reproducibility of computer-aided epicardial and thoracic fat measurement from noncontrast cardiac CT. *J Cardiovasc Comput Tomogr*. 2011;5(3):172-179. doi:10.1016/j.jcct.2011.03.009

10. Goeller M, Achenbach S, Marwan M, et al. Epicardial adipose tissue density and volume are related to subclinical atherosclerosis, inflammation and major adverse cardiac events in asymptomatic subjects. *J Cardiovasc Comput Tomogr*. 2018;12(1):67-73. doi:10.1016/j.jcct.2017.11.007

11. X L, Y S, L X, et al. Automatic quantification of epicardial adipose tissue volume. *Medical physics*. 2021;48(8). doi:10.1002/mp.15012

12. Li X, Ji L, Zhang R, et al. COACT: coronary artery centerline tracker. *Med Phys*. 2024;51(5):3541-3554. doi:10.1002/mp.16873

13. Starekova J, Hernando D, Pickhardt PJ, Reeder SB. Quantification of Liver Fat Content with CT and MRI: State of the Art. *Radiology*. 2021;301(2):250. doi:10.1148/radiol.2021204288

14. Kim DY, Park SH, Lee SS, et al. Contrast-enhanced computed tomography for the diagnosis of fatty liver: prospective study with same-day biopsy used as the reference standard. *Eur Radiol*. 2010;20(2):359-366. doi:10.1007/s00330-009-1560-x
